# Child and Family Adaptation to Juvenile Idiopathic Arthritis—A Systematic Review of the Role of Resilience Resources and Mechanisms

**DOI:** 10.3389/fpsyg.2019.02445

**Published:** 2019-11-05

**Authors:** Lisa Hynes, Sophia Saetes, Brian McGuire, Line Caes

**Affiliations:** ^1^Discipline of General Practice, School of Medicine, National University of Ireland, Galway, Ireland; ^2^School of Psychology and Centre for Pain Research, National University of Ireland, Galway, Ireland; ^3^Division of Psychology, Faculty of Natural Sciences, University of Stirling, Stirling, United Kingdom

**Keywords:** resilience, chronic pain, juvenile Idiopathic arthritis, children, family

## Abstract

**Background:** Juvenile Idiopathic Arthritis (JIA) is the most common rheumatic disease in childhood, with chronic pain being a main symptom. JIA symptoms can lead to substantial disability in children and their families. While preliminary evidence reveals the potential beneficial role of resilience in dealing with chronic pain, research on the role of resilience in how families of a child with JIA cope with pain-related symptoms is scant and dispersed.

**Objectives:** Using the framework of the Ecological Resilience-Risk Model, this review aims to identify (1) family characteristics that are associated with both risk and resilience in children with JIA and (2) the contribution of individual and parental resilience mechanisms and resources to resilience outcomes in children with JIA and their families.

**Methods:** MEDLINE, EMBASE, EBSCO, Psycharticles, and PsycINFO were systematically searched. Longitudinal, cross-sectional, and treatment studies written in English with a focus on resilience resources and/or mechanisms in families of a child (6–18 years) with JIA were included. The original search (July 2016) produced 415 articles, with a final sample of 6 articles remaining after screening. An updated search (July 2018) did not identify new articles, but identified one extra article through personal communications. The 7 articles were included in a narrative review and study quality was assessed.

**Results:** Limited research was available on the role of family characteristics, with just one study revealing how family dysfunction is related to reduced child resilience. Studies evaluating the role of individual resilience mechanisms and resources most commonly assessed resilience outcomes in terms of recovery and sustainability outcomes, such as health-related quality of life (HRQL) and functional disability. The findings revealed that children's psychological flexibility, self-efficacy, adherence, pain acceptance, and perceived social support contribute to resilience outcomes. Findings were inconclusive for the influence of coping strategies, such as seeking social support.

**Conclusions:** While our knowledge is growing, a better understanding of how familial and individual resilience resources and mechanisms influence adjustment to chronic pain as part of JIA is needed and can stimulate development of targeted interventions to enhance outcomes for children with JIA.

## Introduction

Chronic pain, defined as frequent, or recurrent pain that lasts for longer than 3 months (American Pain Society, 2001), is a common condition that occurs regardless of age, sex, or social status (King et al., 2011). In particular, chronic pain is a common symptom of Juvenile Idiopathic Arthritis (JIA), which is the most common rheumatic disease in childhood. JIA is diagnosed in children below 16 years of age when arthritis is identified in at least one joint, for a minimum of 6 weeks (Clinch and Eccleston, 2009; Stinson et al., 2012).

The trajectory of JIA is unpredictable with a wide range of physical (pain, stiffness) and emotional (anxiety, depression) symptoms that can restrict physical and social interactions, thereby potentially inducing functional disability across the lifespan (Sawyer et al., 2005). Indeed, about 30–56% of children with JIA experience continued functional limitations throughout their lifespan (Packham and Hall, 2002). Consequently, a principal aim of multidisciplinary treatment approaches for JIA is to support children in adopting effective coping mechanisms for adjusting to the condition, thereby to facilitating adaptation to JIA (Stinson et al., 2012).

Resilience may be one process that determines whether adjustment difficulties (such as post-traumatic stress symptoms) or positive adaptation (post-traumatic growth) will be observed in response to a major life event. Resilience can be been defined as “*a dynamic and multi-systemic progression that allows the individual to respond effectively when faced with risk or adversity (e.g. medical condition)”* (Cousins et al., 2015). While the process of resilience originates within the individual, social, and environmental factors contribute substantially to the process of resilience. Resilience in the face of a pediatric chronic illness has been operationalized in various ways, all with a focus on demonstrating outcomes such as health-related quality of life (HRQL), in line with or exceeding normative development, despite being faced with managing a chronic illness (Hilliard et al., 2015). Commonly assessed concepts include post-traumatic growth, adaptation, self-esteem, self-concept, optimism, and hope (Cousins et al., 2015).

Within the context of pediatric chronic pain in particular, the recently developed Ecological Resilience-Risk Model (ERRM; Cousins et al., 2015) is based on a growing body of evidence highlighting mechanisms which optimize HRQL in children with chronic pain. The ERRM provides a framework to evaluate the interdependent role of individual and familial resilience and risk factors in adjusting to pediatric chronic pain (Cousins et al., 2015). The ERRM identifies resilience and risk factors as independent but related constructs determining the child's pain trajectory. Importantly, the ERRM framework distinguishes between resilience mechanisms, defined as dynamic, modifiable processes children, or families engage in as a response to pain experiences (e.g., self-efficacy and pain acceptance), vs. resilience resources, defined as stable individual traits, or familial factors (e.g., optimism, and social support),The framework describes and recognize show both child and parent resilience resources and resilience mechanisms interact to promote resilience outcomes. Resilience outcomes are further categorized as recovery and sustainability (i.e., continued or resumed engagement with daily and valued actives, often assessed in terms of HRQL, and academic success) and growth (i.e., enhanced understanding of their capability, often assessed in terms of benefit finding and posttraumatic growth) (Sturgeon and Zautra, 2013; Cousins et al., 2015; Caes et al., 2018). Risk factors, such as negative affect and poor parental health, and risk mechanisms, including catastrophic thinking, and parental overprotective responses are described in the model as forces that can interfere with resilience resources and mechanisms, thereby influencing resilience outcomes. However, the presence of resilience mechanisms and resources can also buffer against the negative impact of risk factors.

Despite the development of the ERRM and the increased research attention on resilience mechanisms, the available evidence is scattered and many of the relationships suggested in the model are yet to be evaluated in the literature. For instance, the ERRM suggests that family context is an important determinant of a child's resilience outcomes. However, most research exploring the role of family resilience focuses on parental responses to pain, such as parent's psychological flexibility (Caes et al., 2018). While important, such a focus lacks the recognition that families are more than the sum of their parts (Mehta et al., 2009). To gain a true understanding of the role of family resilience on how children deal with chronic pain input from all parties involved (i.e., child, parents, and siblings) on family processes, is required.

To guide future research related to supporting resilience in families living with JIA, it is important to clarify the relationships within the ERRM that have and have not been examined in the literature. This review aims to (1) identify family characteristics that are associated with both risk and resilience in children with JIA and (2) identify the contribution of individual and parental resilience mechanisms and resources to resilience outcomes in children with JIA and their families, using the resilience-risk model for pediatric chronic pain (Cousins et al., 2015) as an organizing framework. By synthesizing existing evidence using the ERRM as an organizing framework, researchers, and intervention developers will be in a position to address gaps in the evidence to support further research into the design of effective resilience-focused interventions. The findings will be organized and discussed according to individual and parental resilience and risk resources and mechanisms to delineate the independent and overlapping adjustment and adaptation experiences.

## Method

### Systematic Review Protocol

This systematic review of the current evidence on family resilience in JIA, was conducted and reported in accordance with the Preferred Reporting Items for Systematic Reviews and Meta- Analyses (PRISMA) statement. The protocol for this review is registered with the International Prospective Register of Systematic Reviews (PROSPERO) database (registration number: CRD42016047226; Saetes et al., 2017).

### Search Strategy

A systematic search was conducted in MEDLINE, EMBASE, EBSCO, Psycarticles, and PsycINFO, using the following search terms: (Juvenile Idiopathic Arthritis or JIA or rheumatoid arthritis or systemic-onset Juvenile Idiopathic Arthritis or psoriatic arthritis or enthesitis- related Juvenile Idiopathic Arthritis or oligoarthritis or polyarthritis), (Chronic pain or recurrent pain or pain), (Children or child or adolescence or adolescent or pre adolescence or pediatric or pediatric), (Sibling or family or family function or parent or parenting or parental or peer relationships), (Resilience or resiliency or post traumatic growth or optimism or benefit seeking or benefit finding or coping skills or coping or adjustment or adaptation or health behavior or health behavior or quality of life or hope or psychological resilience or psychosocial functioning or social support or self-concept or acceptance or self- efficacy or positive affect). Other potential sources of relevant literature, known as gray literature, was also reviewed, for example reference sections of relevant publications and conference abstracts.

### Study Selection

Studies were included if they were longitudinal, cross-sectional, or treatment studies; written in English; involved young people aged 6–18 years, with a diagnosis of JIA, who were currently undergoing treatment, and were experiencing chronic pain. Studies meeting these inclusion criteria were included in the review regardless of their gender, arthritis type, and type of treatment. Studies were also included if siblings, aged 6–18 years, and parents were part of the study sample. Studies were excluded for the following reasons: review study; full text was not available; evaluation of measurement tools; sample not living with JIA; not a research study; sample outside the age range; or resilience not measured.

Studies identified by the search strategy were exported to an Endnote database for independent review by two of the authors (LH & SS). All results were reviewed by one author (SS), and 20% were reviewed by another author (LH). Duplicates were removed and studies that did not meet the inclusion criteria were excluded in three phases: review of titles, abstracts, and full texts. After each stage of review, the two authors compared decisions and discussed disagreements until agreement was reached. A third author (LC) was consulted regarding disagreements when an additional perspective was required.

### Data Extraction

Based on the review aims, a data extraction table was created to guide the systematic and standardized extraction of data from included studies. Data extraction was completed by the same two authors responsible for the search strategy. Data were extracted related to the year of publication, journal, database, sample (sample size, demographic information for child with JIA, parents & siblings), methodological aspects (study design, analysis, and measurement tools), resilience resources and mechanisms (e.g., pain acceptance, social support), findings related to the impact of resilience in families, and study limitations. The last author (LC) reviewed the extracted information to confirm the accuracy and adequacy of the data extraction process.

### Quality Appraisal

The studies included in this review were appraised for quality using a method developed and utilized in a similar systematic review (Alderfer et al., 2010). For the purposes of their review, Alderfer et al. (2010) developed a 9-criteria appraisal tool with a 3-point rating scale, based on published recommendations. The criteria are: explicit scientific context & purposed; methods used; measurement reliability & statistics; statistical power; internal validity; measurement validity; external validity; appropriate discussion; contribution to knowledge. Based on reports under each criterion, included studies are rated as low (1), medium (2), or high quality (3).

### Data Synthesis

A narrative synthesis of the findings extracted from the studies included in this review was chosen as the most appropriate method of analysis. This method for analyzing the findings of systematic reviews aims to identify themes and patterns across studies to present an overview of the evidence, which goes beyond description of the individual studies (Popay et al., 2006). The included studies approached the conceptualization and measurement of risk and resilience factors related to adaptation to JIA in different ways, using different designs. Therefore, meta-analysis of quantitative findings was not possible. Guidelines on narrative synthesis were used to guide the organization, analysis, and reporting of the findings in this review (Popay et al., 2006). The findings of included studies related to the review aim were summarized in the data extraction table. Using the Ecological Resilience-Risk Model (Cousins et al., 2015) as a framework, the findings were categorized. Similarities and difference across studies, and patterns of relationships between resilience resources and mechanisms, and resilience outcomes were identified and described.

## Results

### Study Selection and Characteristics

**Figure 1:**
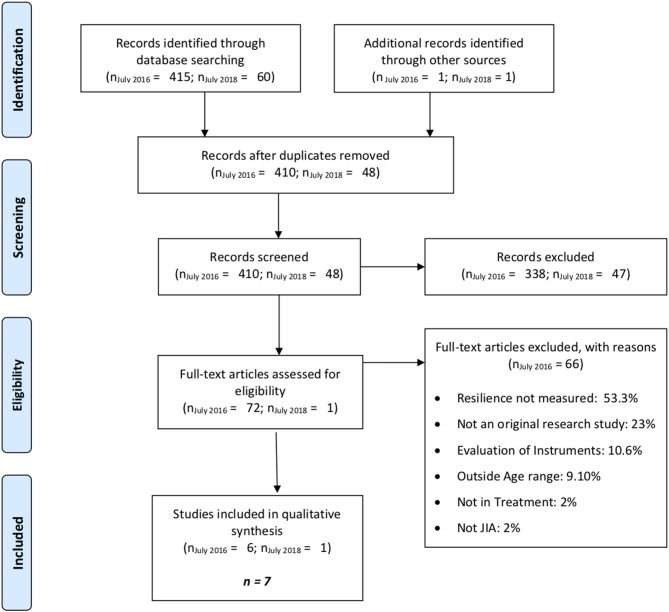
PRISMA flow diagram of study selection.

Databases were first searched in July 2016, and 414 results were retrieved and added to the Endnote database for review. The PRISMA flowchart (Figure 1) presents the results of each stage of the systematic review process. After duplicates were removed, 410 results remained. As a result of review of titles and abstracts, 338 manuscripts were excluded, leaving 72 manuscripts for full text review. Comparisons of review decisions made by the two authors showed high levels of agreement (98% for title and abstract review; 100% for full text review). Review of full texts resulted in the exclusion of 66 manuscripts and six manuscripts to be included in the analysis. The search strategy was conducted again in July 2018 but no newly published literature was eligible for inclusion in this review. However, one additional eligible manuscript was identified through another source (personal communication) in July 2018. In sum, of the 458 studies screened, seven met the inclusion criteria and were analyzed in a narrative synthesis (Timko et al., 1993; Frank et al., 1998; Sawyer et al., 2004, 2005; Connelly, 2005; Seid et al., 2014; Beeckman et al., 2018). Table 1 provides details on study design, samples, measurement tools, and quality.

**Table 1:** Characteristics of studies.

**References**	**Country**	**Design**	**Sample size (% female) Mean age of pediatric sample (SD)**	**Resilience resources & mechanisms are measured**	**Outcomes related to review aims**	**Quality appraisal rating**
(Beeckman et al., 2018)	Belgium	Cross-sectional questionnaire study	59 (61%) 13.76 year (2.67)	*Resources:* Positive & negative affect	Individual and parent resilience mechanisms directly and indirectly associated with resilience outcomes (QoL/functioning, mood/affect), and can buffer to reduce risk associated with pain intensity.	2
				*Mechanisms:* Child general psychological flexibility and pain acceptance, parent general and pain related psychological flexibility		
(Seid et al., 2014)	United States	Prospective longitudinal cohort study	230 (69.1%) 9.42 year (4.49)	*Resources:* Social support, family climate & relationships	Proxy report HRQOL was explained by family risk mechanisms, while self-reported HRQOL was strongly predicted by family/social resilience resources and individual resilience mechanisms.	2
				*Mechanisms:* Symptom-related self-efficacy, parental distress, coping strategies		
(Connelly, 2005)	United States	Cross-sectional questionnaire study	47 (69%) 9.8 year (1.72)	*Resources:* Family functioning, Hope	No relationship between individual or family resilience resources and resilience outcome of recovery/sustainability.	3
				*Mechanisms:* None		
(Sawyer et al., 2005)	Australia	Prospective longitudinal study	54 (57.4%) 12.8 year (3.3)	*Resources:* None	Use of individual resilience mechanisms (child pain coping strategies), have a significant impact on resilience outcome, QoL (Recovery/sustainability), but not always positive. Coping does not appear to mediate between child's experience of pain and HRQL.	2
				*Mechanisms:* Coping strategies		
(Sawyer et al., 2004)	Australia	Cross-sectional questionnaire study	59 (59.3%) 12.6 year (3.3)	*Resources:* None	Use of more positive individual resilience mechanisms (i.e., child pain coping strategies) was associated with better resilience outcomes (QoL—Recovery/sustainability), according to parents and children.	2
				*Mechanisms:* Coping strategies		
(Frank et al., 1998)	United States	Longitudinal cohort study	27 (70.4%) 5.52 year (4.48)	*Resources:* Family adaptability and cohesion, child functioning	Parental risk mechanisms associated with child resilience outcomes (recovery/sustainability)	2
				*Mechanisms:* Parental distress, parental coping strategies		
(Timko et al., 1993)	United States	Longitudinal cohort study	172 (64.5%) 12.6 year (not reported)	*Resources:* Social & community support	Family (mother and father distress) risk mechanism associated with poorer resilience outcomes	2
				*Mechanisms:* Positive social interaction, coping strategies		

The Ecological Resilience-Risk Model (Cousins et al., 2015) was used as a framework for examining and categorizing the findings of the studies included in this review, with respect to the two primary research questions: (1) identify family characteristics that are associated with both risk and resilience in children with JIA and (2) identify the contribution of individual and parental resilience mechanisms and resources to resilience outcomes children with JIA and their families?

The majority of the findings extracted from the included papers related to the contribution of resilience to outcomes in children with JIA and their parents. Siblings were not included as participants in any of the studies reviewed. Therefore, no conclusions could be made on sibling resilience or the impact of resilience on outcomes such as quality of life in this group.

### Quality Appraisal

The quality of the studies included varied, each having strengths and weaknesses that affect the overall quality of this review. Overarching weaknesses include generally homogenous samples, over-reliance on parent-proxy reports, and cross-sectional designs. General strengths of the individual studies include use of validated measures, some longitudinal designs (see e.g., Timko et al., 1993; Frank et al., 1998; Seid et al., 2014) and successful recruitment of fathers as well as mothers (see e.g., Timko et al., 1993), see Table 1 for the quality assessment for each included study. In relation to this review, the most significant challenges to drawing overarching conclusions from the body of literature related to a lack of uniformity across the studies in terms of the variables measured and reported, different study designs, and differences in demographic and disease-related variables reported.

### Narrative Synthesis Findings

Only one study (Connelly, 2005) reported on possible associations between family characteristics and risk or resilience in children with JIA. According to Connelly (2005), there was a negative association between family risk factors and child resilience resources. This cross-sectional study of 68 children with Juvenile Rheumatoid Arthritis (JRA), and their parents assessed family functioning (parent-proxy report, pediatric quality of life (child self-report and parent-proxy report), and children's hope (child self-report). Higher levels of parent-reported family dysfunction were significantly associated with lower levels of child-reported hope among children (*r* = −0.35, *p* < 0.05).

**Figure 2:**
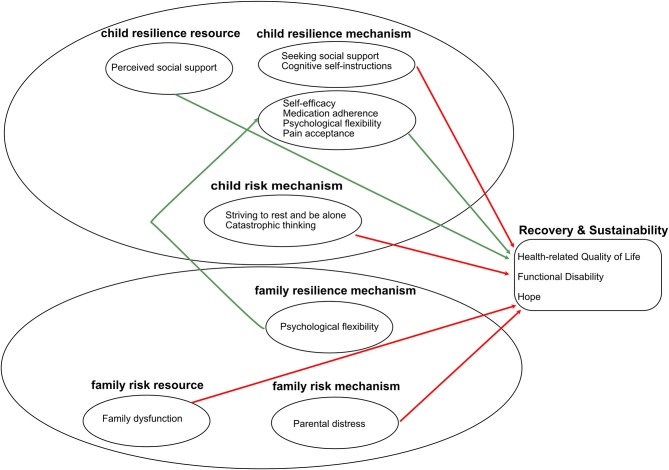
Summary of the findings, using the Ecological Resilience-Risk Model as a framework. Red arrows represent negative associations, green arrows represent positive associations.

In relation to the second primary research question, the findings of this review provide some tentative support for aspects of the Ecological Resilience-Risk Model (Cousins et al., 2015), see Figure 2 for a full overview. The findings suggest that child and parent resilience and risk mechanisms which were measured considerably more frequently than resources and risk factors, may influence resilience outcomes. The most common resilience outcomes measured in the studies included in this review can be characterized as recovery and sustainability outcomes, namely, HRQL and functional disability.

## Resilience Mechanisms

Resilience mechanisms are generally active and dynamic cognitions and behaviors, enhanced by resilience resources, such as social support, which overcome risk factors, and risk mechanisms (Cousins et al., 2015). In this review, three studies (Sawyer et al., 2004; Seid et al., 2014; Beeckman et al., 2018) reported positive relationships between resilience mechanisms, such as coping and psychological flexibility, and outcomes. All three studies included data from parents and children with JIA; however, the Beeckman et al. (2018) and Seid et al. (2014) studies collected data on parent resilience mechanisms, while Sawyer et al. (2004) focused on parent-proxy measurement of variables related to their child's resilience and outcomes.

Sawyer et al. (2004) examined child and parent reports of the use of different pain coping strategies. According to parent-proxy reporting in this study, positive pain coping strategies such as *problem solving/self-efficacy* were associated with less functional disability. As illustrated in the next section, coping strategies acted as risk mechanisms more frequently than resilience mechanisms.

According to Beeckman et al. (2018), child and parental psychological flexibility may support adaptive functioning JIA. Children's general psychological flexibility and pain acceptance were significantly associated with functional outcomes. For example, child psychological flexibility was associated with better psychosocial health (PedsQL emotional, social, and school functioning) (*B* = 0.34, *p* < 0.01) and less negative affect (*B* = −0.60, *p* < 0.01), but was not associated with better physical health or higher levels of positive affect. Child pain acceptance appeared to play an importance role in relation to resilience outcomes, including better psychosocial (*B* = 0.31, *p* < 0.05) and physical health (*B* = −0.45, *p* < 0.001) and less negative affect (*B* = −0.32, *p* < 0.05). Higher levels of pain intensity was associated with disability in this study. Higher levels of child pain acceptance, but not psychological flexibility, appeared to act as a resilience mechanism, and was associated with lower risk of disability among children with high pain intensity in this study.

The potential impact of parent resilience mechanisms on child resilience outcomes was also demonstrated in this study. Although direct effects of parent general and pain-related psychological flexibility were not found, both parent and child resilience mechanisms were indirectly related to better child resilience outcomes. For example, parent's general psychological flexibility was significantly associated with their children's psychological flexibility, which was associated with better psychosocial outcomes and positive affect. Thus, these findings underscore the complex interrelations between parent and child resilience resources and mechanisms, with parent resilience mechanisms influencing child resilience outcomes, via their association with children's resilience mechanisms (flexibility) and resources (affect) (Beeckman et al., 2018).

The focus of the study by Seid et al. (2014) was the predictive ability of non-medical variables (e.g., coping and parental distress) in relation to HRQL. The findings suggest that children rated the impact of non-medical variables on HRQL as being greater than parent-rated impact. For child self-reported HRQL, self-efficacy and adherence to medication made a positive significant contribution. Perceived social support was also be positively associated with child self-reported HRQL, and is one of the few resilience resource variables measured by studies in this review. A significant association was also reported between adherence to medication and HRQL according to parental-proxy measures.

## Risk Mechanisms

Family and individual risk mechanisms were examined in five studies included in this review (Timko et al., 1993; Frank et al., 1998; Sawyer et al., 2004, 2005; Seid et al., 2014). According to the ERRM, risk mechanisms interfere with the pathway to adaptation involving resilience resources and mechanisms, and are enhanced by risk factors.

This review suggests that parental distress is a family risk mechanism that may negatively affect child resilience outcomes (Timko et al., 1993; Frank et al., 1998; Seid et al., 2014). In one study, parental emotional distress was associated with poorer parent-proxy reported child HRQL (Seid et al., 2014). Frank et al. (1998) reported that parental distress at baseline was significantly associated with adaptation to JIA, assessed based on number of swollen joints over 18 months, an indicator of the activity or status of JIA. Higher levels of parental distress in this study were associated with a higher number of swollen joints, suggesting that parental distress may act as a risk mechanism, hindering child adaptation to JIA. Similarly, according to Timko et al. (1993), distress in mothers and fathers was associated with higher levels of functional disability in children.

Three studies (Sawyer et al., 2004, 2005; Seid et al., 2014) reported findings suggesting negative relationships between coping strategies and outcomes. The findings of this review suggest that, when examined in detail, coping strategies frequently act as risk mechanisms. This review also demonstrates considerable disagreement between parents and children in assessments of child coping strategies for JIA.

Seid et al. (2014) reported that, according to child self-report, the use of the coping strategy “catastrophizing” by children, had a significant negative relationship with HRQL. Parental distress and report of use of catastrophizing by their children was also reported to have a significant negative relationship with child HRQL.

According to parent-proxy reporting in the Sawyer et al. (2004) study, higher usage of some pain coping strategies such as *strive to rest and be alone* are associated with poorer child HRQL. Parents in this study rated problem-solving/self-efficacy as the most common pain coping strategy used by their children. However, parents in the study by Sawyer et al. (2005) identified “seeking social support” and “striving to rest and be alone,” as the most frequently used coping mechanisms by their children. According to parent-proxy reports, both of these pain coping mechanisms are associated with poorer physical and emotional functioning. A significant negative association was also found between parent-proxy reports of child pain coping (*seeks social support* and *striving to rest and be alone*) and the daily activities and treatment subscales of HRQL (Sawyer et al., 2005).

In contrast with parent-proxy reports of child coping, children themselves rate cognitive self-instruction (e.g., a child resilience mechanisms in which children imagine they are not in pain, or use positive self-talk related to response to pain) as the most frequently used strategy to cope with pain. Sawyer et al. (2004) reported only significant negative associations between child self-reported pain coping strategies and HRQL. For example, cognitive self-instruction, a child resilience mechanism, was associated with poorer physical functioning. Similarly, seeking social support, also considered a child resilience mechanism, was associated with poorer emotional and social functioning, as well as lower scores on the daily activities and disease-specific treatment subscales. In their 2005 study, Sawyer described similar significant negative associations between child self-reported pain coping strategies and HRQL. In comparison to parent-proxy reporting in this study, negative associations were more commonly reported by children in this study. However, after controlling for pain intensity ratings, the most consistent relationship was found with seeking social support, which was negatively associated with almost all aspects of HRQL, including daily activities, treatment, worry, and physical, emotional, and social functioning.

## Discussion

Using the ERRM for pediatric chronic pain (Cousins et al., 2015) as an organizing framework, this review suggests that both individual and family mechanisms are important in determining outcomes and that parents and children are having different experiences in relation to adaptation to JIA The findings are mostly in accordance with the assumptions of the ERRM and can be summarized as follows. Limited evidence was identified with respect to possible relationships between family characteristics and resilience outcomes. The one study in the review that did address this study aim reported an association between family dysfunction and lower child resilience resources. With respect to resilience mechanisms and resources, the findings provide evidence for significant contributions of several child resilience mechanisms (i.e., self-efficacy, psychological flexibility, pain acceptance, and medication adherence) and resources (i.e., perceived social support) in explaining recovery and sustainability (i.e., HRQL and functional disability). With respect to the influence of child risk mechanisms, evidence was mixed but support was found for children's levels of catastrophic thinking and adopting striving to rest and be alone as a coping mechanism. Furthermore, the results provide support for the important role of family resilience mechanisms (i.e., parental psychological flexibility) and risk mechanisms (i.e., parental distress) as significant contributors to their child's recovery and sustainability. However, different patterns of relationships were identified depending on who (parent proxy report vs. child self-report) was reporting on children's HRQL. This lack of agreement or overlap in parent proxy or child self-report is not unique, and often identified within the literature on children's HRQL. Indeed, parent and child agreement in relation to HRQL is moderate to low and tends to diminish as children age (Rajmil et al., 2013). The complexity of HRQL-reporting illustrated in this review and other previous research emphasizes the importance of gathering data from a range of members of a family unit to understand family resilience in the face of JIA (Mehta et al., 2009). Furthermore, most studies did not look at the full range of potential resilience outcomes but were restricted in relying on HRQL as a resilience outcome. As a result, the big picture in terms of the individual and interacting roles of resilience and risk factors in influencing resilience outcomes cannot be concluded from any one study in this review. Nevertheless, some interesting patterns of key resilience resources and mechanisms were identified.

The findings highlight that parental distress in response to child pain experiences in the context of JIA can be considered a family risk mechanism that negatively impacts child resilience outcomes. Within the broader pediatric chronic pain literature, the recent review by Palermo et al. (2014) summarizes a substantial body of evidence supporting the interrelation between child pain experiences and parental distress. For instance, this review by Palermo et al. (2014) highlights that a considerable number of parents of children with chronic pain experience clinically relevant levels of distress, which has been shown to be negatively related to child pain outcomes, such as increased pain intensity, disability, and distress (Palermo et al., 2014). The findings from our systematic review add to this growing literature by identifying that parental distress also represents a risk factor to children's broader adjustment to JIA by negatively impacting child HRQL, number of swollen joints, and functional disability. Such evidence emphasizes the need to actively involve parents within multidisciplinary treatment approaches for JIA. Providing parents with adaptive coping mechanisms may support them to deal with their own emotional difficulties in response to their child's diagnosis of JIA and associated symptoms. Several parent-focused interventions have been developed in the context of pediatric chronic pain, such as parental problem solving skills training (Palermo et al., 2016). Use of the ERRM for pediatric chronic pain (Cousins et al., 2015) to optimize and integrate such treatment approaches within the care plan for families of a child with JIA may play an important role in supporting child and family resilience. Our findings highlight that the ERRM can be a useful framework to guide such intervention development because it takes the individual and family into account, as well as stable and dynamic characteristics and processes relevant to promoting resilience in the context of JIA.

Although a wide range of evidence was obtained and analyzed with respect to child resilience mechanisms and resources, studies evaluating the role of child coping mechanisms provided equivocal findings. In particular, the coping strategies “seeking social support” and “cognitive self-instructions,” typically considered adaptive coping strategies or resilience mechanisms, were found to be related to poorer functioning and HRQL in this review. A potential explanation for these unexpected findings could be that while, in principle the coping strategies are considered to be adaptive or promoting resilience, the specific relation to child's HRQL does depend on the child's developmental age and the exact way the child is engaging with this strategy. Although evidence indicates that positive peer relationships can strengthen perceived social competence and development in children with chronic pain (Forgeron et al., 2011), not all children's peer relationships are necessarily of a positive and supportive nature. Consequently, the social support a particular child receives might be dependent on the particular characteristics of each relationship, the child's context, level of adjustment or adaptation to JIA, and support needs at any one time. Similarly, to assess the effectiveness of cognitive self-instruction among children, focusing on frequency of cognitive self-instruction alone is insufficient, and the child's capacity to engage positively in this coping mechanism must also be known. These findings with respect to role of coping further support the call made by Van Damme et al. (2008) to step away from traditional categorical approaches toward pain coping strategies (i.e., adaptive vs. maladaptive coping) and adopt a motivational approach. This proposed motivational approach focuses on evaluating the function of each coping strategy in its particular context to determine to what extent the coping mechanism does or does not facilitate adaptation for each individual. Consequently, coping strategies are not inherently a resilience or risk mechanism, but their adaptive function depends on the extent the coping mechanism promotes resilience for a particular child given their unique situation and developmental capacities. Adopting such a motivational perspective on coping within the ERRM framework could allow for better insight into how child's resilience mechanisms, such as coping strategies, develop, and thereby provide more clarity on their complex impact on resilience outcomes. Such insights have the potential to guide the generation of interventions aimed at promoting resilience in a personalized and developmentally appropriate manner.

The conclusions from this review need to be interpreted in light of several limitations. Importantly, drawing strong conclusions is hampered by the limited evidence identified to include in this systematic review. Furthermore, the quality assessment of the studies included in this review highlighted issues and variations in methodological quality. In particular, the majority of the studies based their findings on cross-sectional designs in homogenous samples and were overly reliant on parent-proxy reports. Importantly, no studies were identified for inclusion in the review that investigated the impact of JIA on siblings and the role of siblings' resilience resources and mechanisms. Therefore, to expand our understanding of family resilience in the context of JIA, future studies must examine important variables from the perspectives of multiple family members, including children with JIA and their siblings. Research that is longitudinal in nature and includes heterogeneous populations are called for. Furthermore, we could only include quantitative studies in this review. A more complete understanding of the development and influence of resilience resources and mechanisms will be made possible by the use of different approaches, including more intervention research, research using mixed methods and qualitative approaches.

Despite these limitations, the findings do provide preliminary insight into the application of the ERRM to understand which individual and family psychological processes may influence resilience in children with JIA. Given the limited empirical evidence for these resilience mechanisms and resources in the broader literature on pediatric chronic pain, a similar review with respect to resilience in the context of any pediatric chronic pain experience might be warranted to move this field forward. Our findings also highlight some limitations and challenges of the ERRM. In particular, the distinction between resilience mechanism and outcomes is not always straightforward and mostly depends on the specific operationalisation of these constructs within a study. For instance, Beeckman et al. (2018) considered affect to be a resilience outcome, whereas this is considered a resilience resource according to the ERRM. Similarly, Seid et al. (2014) considered adherence or self-management behaviors as resilience mechanisms, while appropriate adherence could also be a sign of having adapted to life with JIA and hence represent a resilience outcome. More theoretical developmental research is needed to enhance our understanding of these resilience pathways in the context of childhood chronic illness. As the ERRM framework was developed based on an adult framework, it is possible that these distinctions between mechanisms and outcomes are clearer in adulthood but are less distinct in childhood. Furthering of our theoretical understanding of family resilience and the role of both individual and familial resilience pathways is needed to inform the development and refinement of targeted interventions to enhance clinical practice and interventions aimed at fostering resilience in all family members of a child diagnosed with JIA.

## Author Contributions

LH assisted SS in the study selection and data extraction. In the write up of the manuscript, LH took charge of writing up the methods and results, and provided feedback on the introduction and discussion. SS took charge of designing the systematic review, the study selection process and data extraction and wrote a first draft of the methods and results section, which was used by LH to write up these sections, and also provided feedback on the full version of the manuscript. BM supervised the study selection and data extraction, conducted by SS and LH and provided feedback on the full version of the manuscript. LC supervised the study selection and data extraction, conducted by SS and LH. LC was actively involved in the write up of the data, taking charge of writing the introduction and discussion, as well as providing feedback on the write up of the methods and the results.

### Conflict of Interest

The authors declare that the research was conducted in the absence of any commercial or financial relationships that could be construed as a potential conflict of interest.
